# ‘Dumpling suture method’ versus traditional suture method of protective loop ileostomy in laparoscopic anterior rectal resection with specimen extraction through stoma incision: a retrospective comparative cohort study

**DOI:** 10.1097/JS9.0000000000000953

**Published:** 2023-12-04

**Authors:** Jiani Gu, Jin Wang, Xingwang Hu, Wenjun Ding, Long Cui, Peng Du, Zhonglin Liang, Tingyu Wu

**Affiliations:** Department of Colorectal and Anal Surgery, Xinhua Hospital, Shanghai Jiao Tong University School of Medicine, Shanghai, People’s Republic of China

**Keywords:** diverting loop ileostomy, dumpling suture method, laparoscopic anterior rectal resection, rectal cancer, stoma-related complication

## Abstract

**Background:**

A diverting loop ileostomy (DLI) is performed in laparoscopic anterior rectal resection (LAR) surgery at high risk of anastomotic fistula. Minimally invasive surgery promotes postoperative recovery and cosmetics. To reduce abdominal trauma, specimen extraction through stoma incision (EXSI) is usually performed to avoid auxiliary abdominal incision with enlarged stomal incision. The traditional suture method (TSM) reduces the incision size by suturing the ends of the enlarged incision, leading to peristomal incisions and a higher risk of stomal complications. The study aimed to introduce the dumpling suture method (DSM) of PLI and compare this new method with TSM.

**Materials and Methods:**

The authors propose a novel stoma suture technique, which utilized a method of skin folding suture to reduce the enlarged incision size. A retrospective analysis was conducted on 71 consecutive patients with rectal cancer who underwent LAR-DLI with EXSI, and the intraoperative details and postoperative outcomes of the two groups were measured.

**Results:**

The DSM group showed a lower stomal complication rate (10.3 vs. 35.7%, *P*=0.016) than that of the TSM group. The scores of DET (Discoloration, Erosion, Tissue overgrowth), stomal pain, quality of life were all significantly lower in DSM group than in TSM group. In multivariate analysis, DSM was an independent protective factor for stoma-related complications. Operative time, time to first flatus, defecation and eat, nonstomal related postoperative complications were similar in both groups.

**Conclusion:**

DSM utilizes a method of skin folding suture to reduce the enlarged incision size, which is safe and effective in reducing the incidence of peristomal skin infections and stomal complications. This procedure offers a novel suturing approach for loop ileostomy with enlarged incision, effectively reducing the postoperative trauma and incidence of stomal complications.

## Introduction

Highlights

- Specimen extraction through stoma incision leads to stomal complication.
- We propose a novel stoma suture technique, named ‘Dumpling Suture Method (DSM)’.
- DSM utilizes a method of skin folding suture to reduce the enlarged incision size.
- DSM is safe and effective in preventing skin infections and stomal complications.
- DSM offers a novel suturing approach for loop ileostomy with enlarged incision.

In rectal cancer surgery, particularly in patients with low rectal cancer, the incidence of anastomotic fistula ranges from 9.8 to 37.5% in laparoscopic anterior rectal resection (LAR), which represents a significant postoperative concern and often requires a secondary operation^1–4^. Therefore, in patients at high risk for anastomotic fistula, diverting loop ileostomy (DLI) to divert fecal flow is routinely performed, which can protect the anastomosis and reduce the complications caused by postoperative anastomotic leakage^5^. In LAR-DLI, an auxiliary abdominal incision typically measuring 5–10 cm in length is usually made for specimen extraction.

Recent decades have witnessed technological advancements and innovations in colorectal surgery. These developments have not only resulted in a decrease in abdominal surgical incisions, but have also eliminated the need for additional specimen extraction sites. For patients who require a DLI in LAR surgery, many surgeons utilize an enlarged stoma incision for specimen extraction in an attempt to avoid an auxiliary abdominal incision^6^. Following surgery, there is only the stoma in the abdominal wall, without any additional incisions, which promotes postoperative recovery and favorable cosmetics with reduced trauma.

However, this technique is not widely employed in practical applications and associated with considerable stoma complications, which reported to be 13–33%, thereby limiting the potential for broader implementation^6–9^. In our center, the stomal complication rate of LAR-DLI with specimen extraction through stoma incision (EXSI) is 35%. EXSI can result in an oversized incision measuring between 5 and 10 cm. To reduce the incision size and match it to the stoma size, the traditional suture method (TSM) uses interrupted suture at both ends of the incision to reduce the size to 3 cm, followed by fixing the stoma to the skin. TSM results in an additional incision adjacent to the stoma, which can increase the incidence of fecal leakage under the base plate of stoma bag. Direct contact with highly corrosive intestinal fluids often leads to infection of peristomal incision and stoma-related complications including fecal dermatitis, skin erosion, skin ulcer, and mucocutaneous detachment.

In an effort to mitigate stoma-related complications, we are introducing a novel stoma suturing technique. This technique employs a method of suturing the stoma in conjunction with a skin fold, gradually reducing the incision to 3 cm, similar to the process of making Chinese dumplings, which is named ‘Dumpling Suture Method (DSM)’ in our center. Postoperatively, the absence of peristomal incision can effectively reduce the incidence of peristomal skin infections and stoma-related complications.

To assess the efficacy and safety of this new procedure, we report the DSM of DLI in LAR with specimen extraction through stoma incision in surgical picture, illustration and video and compared this method to the TSM in a retrospective comparative cohort study. The trial was registered by the Clinical Trial with a unique identification number.

## Materials and methods

### Study population

**Figure 1:**
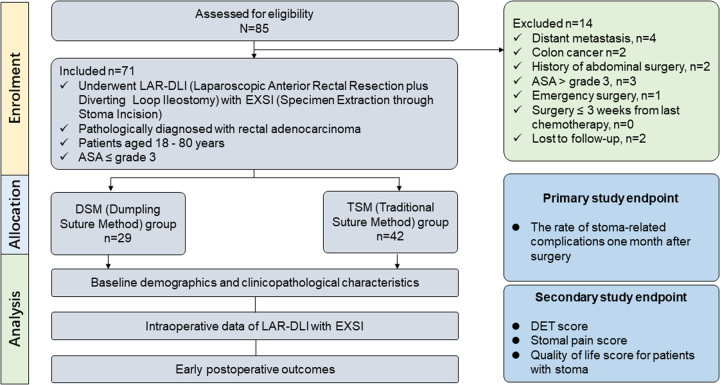
Flowchart and study endpoint of the retrospective cohort study. All study endpoints were assessed at 1 month postoperatively.

We conducted a retrospective study that included a consecutive series of patients with rectal cancer who underwent laparoscopic anterior rectal resection plus diverting loop ileostomy (LAR-DLI) with specimen extraction through stoma incision (EXSI) at a tertiary referral hospital between November 2020 and May 2023.The inclusion criteria were as follows: (1) All patients underwent successful LAR-DLI with EXSI; (2) All patients were pathologically diagnosed with rectal adenocarcinoma; (3) Patients aged 18–80 years, with ASA (American Society of Anesthesiologists) classification ≤ grade 3. Patients with distant metastasis, colon cancer, a history of previous abdominal surgery, ASA classification >grade 3, and those who underwent emergency surgery, underwent surgery ≤3 weeks from the last chemotherapy or lost to follow-up were excluded. Following the application of these inclusion and exclusion criteria, the included patients were divided into two groups: the DSM (Dumpling Suture Method) group and TSM (Traditional Suture Method) group according to the ileostomy suture method (Fig. 1). The choice to use the DSM or TSM for ileostomy was determined individually by different surgeons, based on their familiarity with different procedures and surgical preferences.

### Surgical procedures of LAR-DLI with TSM

To avoid interference with postoperative results by different stoma positioning, the same skilled entero-stomal therapist (ET) preoperatively marked and positioned stoma on patients according to uniform criteria. All of patients underwent standard bowel preparation. Prophylactic antibiotics were administered 30–60 min prior to the initiation of the surgical procedure. Five trocar laparoscopic approach were used.

**Figure 2:**
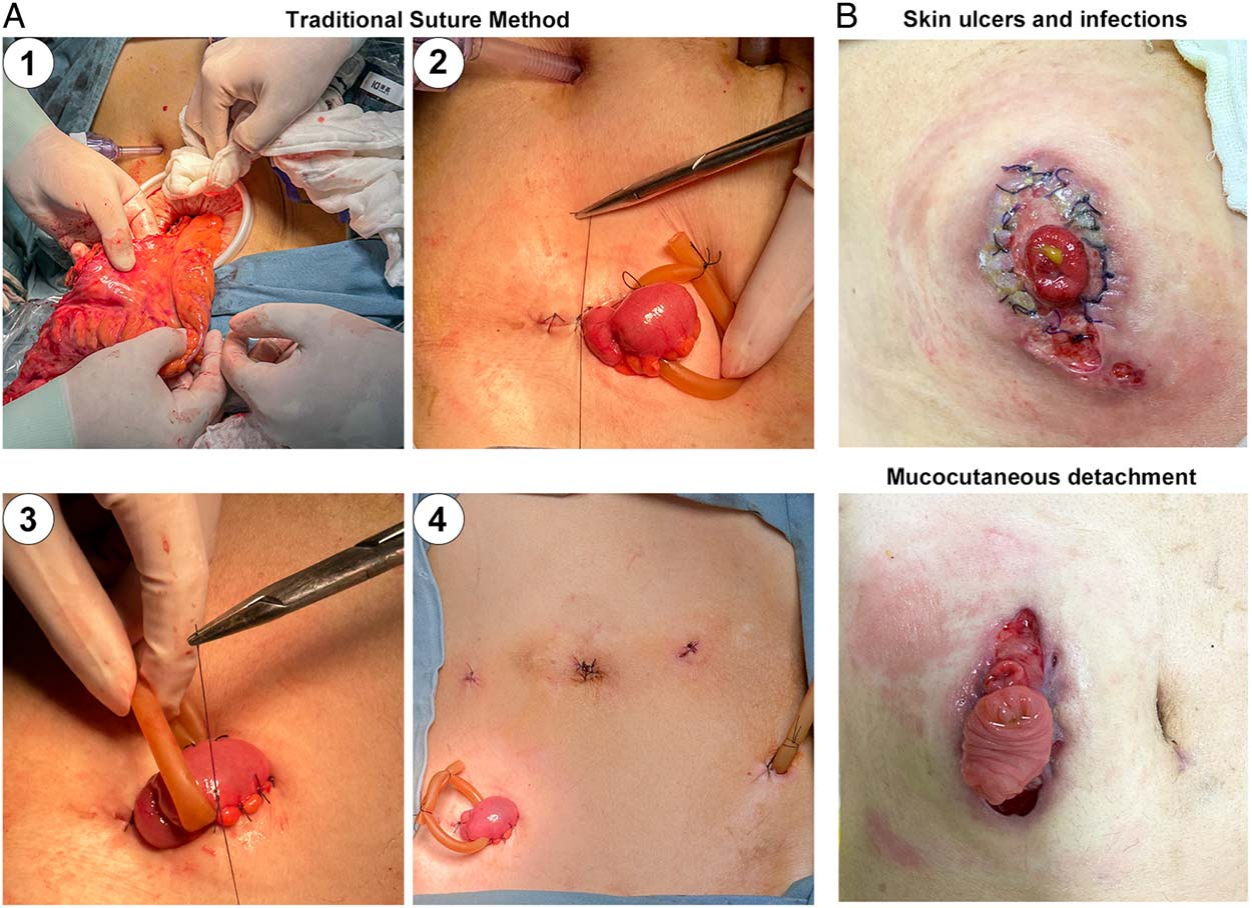
Surgical procedures and complications of traditional suture method of diverting loop ileostomy. (A) Surgical procedures including: 1) The specimen was extracted from the incision of the stoma, rectal tumor was removed intact; 2) Suture both ends or a single end of the incision, thereby reducing the incision size to 3 cm; 3) Fix the stoma to the skin with interrupted sutures; 4) The postoperative abdominal wall only has trocar holes and a stoma with no additional auxiliary incisions. (B) Examples of postoperative stomal complications at 1 month postoperatively.

The surgical procedure of LAR was compiled based on TME. After resection of the proximal rectum in the abdominal cavity, an appropriate stoma position was selected in the lower right abdominal wall within the rectus abdominis muscle to create a matched abdominal incision (5–10 cm) according to the size of rectal tumor. After the incision was made, an 8 cm diameter incision protection sleeve was disposed into the incision to prevent tumor dissemination and incision infection (Fig. 2A).

The specimen was then extracted from the incision of the stoma, rectal tumor was removed intact, the nail seat was placed into the proximal severed end of descending colon. The descending colon is redelivered into the pelvic cavity, pneumoperitoneum was re-established, followed by an end-to-end anastomosis.

To prevent parastomal hernia due to the larger size of the stoma incision (5–10 cm), the peritoneal layer ends and anterior sheath ends of the rectus muscle of the incision were closed with 3-0 absorbable sutures, which reduced the opening of peritoneal layer to about 3 cm. The terminal ileum ~40 cm proximal to the ileocecal junction was chosen as the stoma position. The terminal ileum was extracted using a rubber tube through a small hole in the nonvascular area of the ileal mesentery close to the bowel.

**Figure 3:**
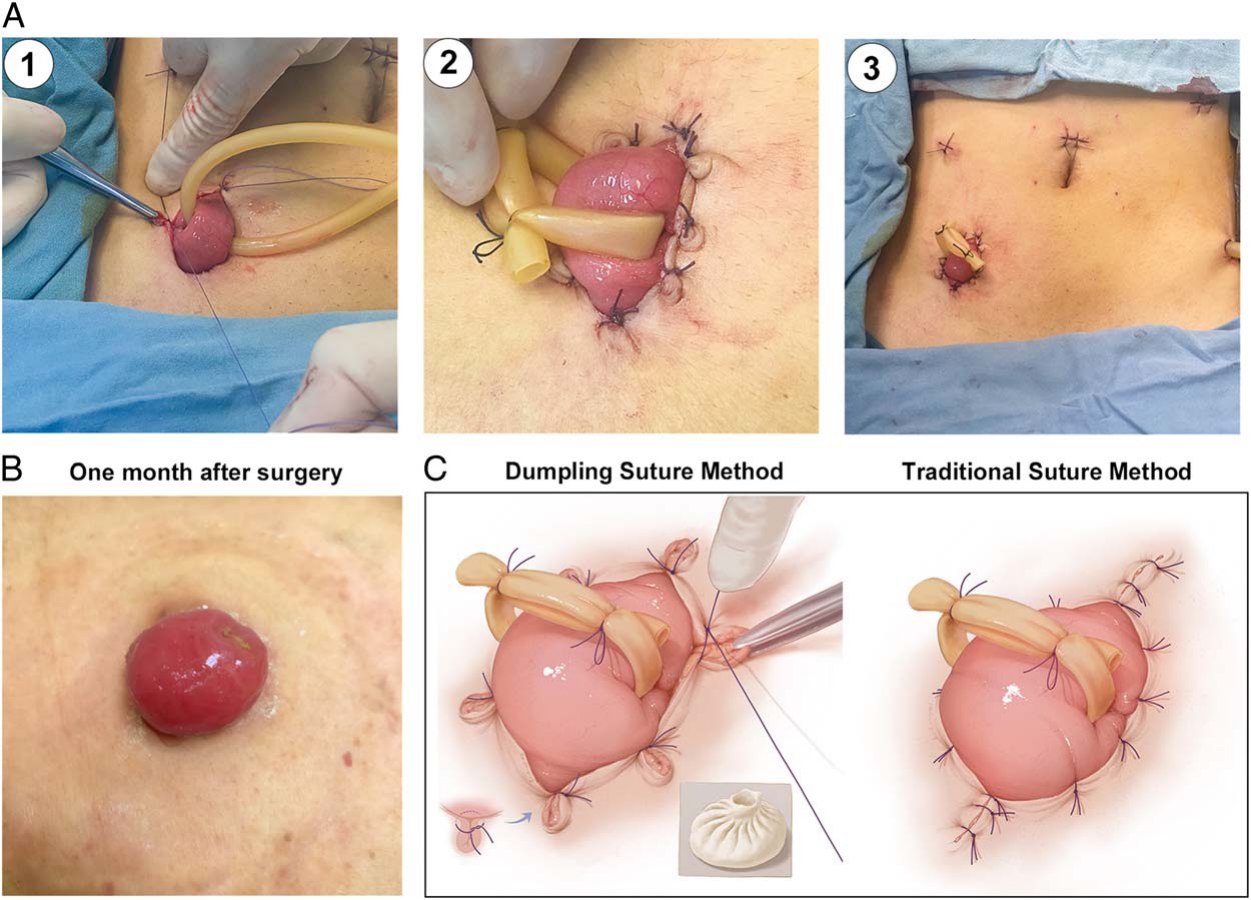
Surgical procedures of ‘Dumpling suture method’ of diverting loop ileostomy. (A) Surgical procedures including 1) 3-0 absorbable suture was employed to pass through the tissues in a sequence of skin-ileum-skin (forming a U-shape), with the U-shaped opening maintained at a width of 3–4 mm. A toothed forceps was employed to elevate the skin edge, creating an outward angle which was maintained. The assistant tied the knot against the side of the intestinal wall. 2) The stoma is fixed to the skin with 8–12 folded sutures. 3) The postoperative abdominal wall only has trocar holes and a stoma with no additional auxiliary incisions. (B) One month after the surgery, the peristomal skin exhibited favorable healing, and no skin folds or infection were detected. (C) Surgical illustration of DSM and TSM.

The enlarged incision is excessively large in relation to the stoma. In order to achieve a suitable alignment between the stoma and the large incision (5–10 cm), TSM for ileostomy was performed to reduce the incision size (Fig. 2A, Fig. 3C): (1) Suture both ends or single end of the incision, thereby reducing the incision size to 3 cm. (2) Then the loop ileum with rubber ring was extracted from the incision, with 2 cm above the abdominal wall. (3) Fix the stoma to the skin with interrupted sutures. (4) Finally, a small incision was made in the intestinal wall to open the stoma. The rubber ring placed around the stoma served to support the stoma and prevent stoma retraction, which was removed within 1–2 weeks postoperatively. The postoperative abdominal wall only has trocar holes and a stoma with no additional auxiliary incisions (Fig. 2A).

### Surgical procedures of DSM

In order to reduce the stoma skin incision, DSM employs a method of fixing the stoma to the skin through the utilization of skin folding sutures, without suturing the incision ends as in TSM, thus avoiding the formation of peristomal incisions. This process of making skin folds is similar to making the folds of Chinese dumplings (Fig. 3C). DSM for ileostomy was as follows (Fig. 3A) (see video in Supplementary Materials, Supplemental Digital Content 1, http://links.lww.com/JS9/B504): (1) The loop ileum with rubber ring was extracted from the incision, with 2 cm above the abdominal wall. (2) 3-0 absorbable suture was employed to pass through the tissues in a sequence of skin-ileum-skin (forming a U-shape), with the U-shaped opening maintained at a width of 3–4 mm. (3) A toothed forceps was employed to elevate the skin edge, creating an outward angle which was maintained. The assistant tied the knot against the side of the intestinal wall. This maneuver ensured the fixation of the protruded portion of the skin edge to the exterior of the suture knot. (4) The stoma is fixed to the skin with 8–12 folded sutures in this way and the goal of reducing the size of the incision is achieved, without the formation of peristomal incisions. (5) Finally, a small incision was made in the intestinal wall to open the stoma. The rubber ring served to support the stoma and prevent stoma retraction, which was removed within 1–2 weeks postoperatively. Peristomal sutures should be removed 2 weeks after surgery.

### Data collection

The trial was registered by the Clinical Trial of U.S. National Library of Medicine with a unique identification number. The study was approved by the hospital ethics committee (XHEC-2023-D-146) and was carried out in accordance with the 1964 Helsinki Declaration. Written informed consent was obtained from the patients. All data were obtained from the patients’ medical records. The baseline demographics, clinicopathological characteristics, intraoperative details and early postoperative outcomes were analyzed. This study was conducted in accordance with the strengthening the reporting of cohort, cross-sectional and case–control studies in surgery (STROCSS) criteria^10^ (Supplemental Digital Content 2, http://links.lww.com/JS9/B505).

### Outcome

The primary study endpoint was the rate of stoma-related complications in the DSM and TSM groups 1 month after surgery. The secondary study endpoint of this study was to investigate DET score, stomal pain score, and quality of life score for patients with stoma 1 month after surgery.

The DET (Discoloration, Erosion, and Tissue overgrowth) score scale serves as an effective tool specifically designed for the assessment of peristomal skin lesion conditions, including the evaluation of skin discoloration, erosion and tissue overgrowth, and translated into an ordinal variable with four categories: None: DET=0; Mild: DET =1-5; Moderate=5–10; Severe=11–15.

The NRS (Numeric Rating Scale) is employed to assess patients’ pain intensity. Self-assessment is performed on a 10-point scale, with degrees ranging from 1 to 10. Pain levels are categorized based on corresponding numbers, including Level 0 for no pain, Levels 1–3 for mild pain, Levels 4–6 for moderate pain, and Levels 7–10 for severe pain.

Patient quality of life assessments were conducted utilizing COH-QOL-OQ (City of Hope Quality of Life-Ostomy Questionnaire), a specific scale designed for ostomy patients. Patients provided ratings based on the impact of the ostomy on physical, psychological, and social well-being. With a maximum score of 430, higher scores indicate a more pronounced detrimental effect of the ostomy on quality of life.

### Statistical analysis

Student’s *t*-test and Mann–Whitney *U* test were used for quantitative variables with normal and non-normal distribution. Pearson’s χ^2^ test and Fisher’s exact test were used for nominal variables. A multivariable logistic regression model was used to evaluate the risk of stoma-related complications, adjusting for covariates determined *a priori* to be clinically relevant. These covariates included age, sex, ASA class, BMI, combined diabetes, hypoalbuminemia, neoadjuvant therapy, initial incision length, and surgical methods. Statistical analysis was performed using SPSS 27 software. Statistical significance was defined at *P*-values<0.05.

## Result

### Baseline demographics and clinicopathologic characteristics

A total of 85 rectal adenocarcinoma patients who underwent LAR-DLI with specimen extraction through stoma incision (EXSI) from November 2020 and May 2023 were screened for the study; of these patients, 14 were excluded (distant metastasis, *n*=4; colon cancer *n*=2; history of abdominal surgery, *n*=2; ASA >grade 3, *n*=3; emergency surgery, *n*=1; lost to follow-up, *n*=2) (Fig. 1). A total of 71 patients who met the study criteria were enrolled in this study [53 men (53.5%)] (Table 1). Twenty-nine patients underwent DLI by the DSM, while 42 patients underwent surgery with the TSM. The baseline demographics and the clinicopathological characteristics are summarized in Table 1. No significant differences were detected between the DSM and TSM groups with respect to age, sex, BMI, ASA class, current smoker, combined diabetes, hypoalbuminemia, neoadjuvant therapy, preoperative intestinal obstruction, distance of tumor from anal verge, tumor size, surgical technique and pathological TNM stage.

**Table 1:** Baseline demographics and clinicopathologic characteristics of the patients.

	Patients undergo LAR-DLI (*n*=71)		
Variable	DSM (*n*=29)^a^	TSM (*n*=42)^a^	*P*
Sex			0.474^b^
Male	17 (58.6%)	21 (50%)	
Female	12 (41.4%)	21 (50%)	
Age, years	58.2 (14.1)	59.9 (12)	0.592^c^
BMI, kg/m2	23.4 (2.2)	23.1 (3.1)	0.577^c^
Current smoker within 1 year			0.624^b^
Yes	3 (10.3%)	6 (14.3%)	
No	26 (89.7%)	36 (85.7%)	
Combined diabetes			0.953^b^
Yes	4 (13.8%)	6 (14.3%)	
No	25 (86.2%)	36 (85.7%)	
ASA			0.317^b^
I	5 (17.2%)	14 (33.3%)	
II	10 (34.5%)	11 (26.2%)	
III	14 (48.3)	17 (40.5)	
Hypoalbuminemia			0.814^b^
Yes	4 (13.8%)	5 (11.9%)	
No	25 (86.2%)	37 (88.1%)	
Neoadjuvant therapy			0.656^b^
Yes	9 (31%)	11 (26.2%)	
No	20 (69%)	31 (73.8%)	
Preoperative Intestinal obstruction			0.787^d^
Yes	1 (3.4%)	2 (4.8%)	
No	28 (96.6%)	40 (95.2%)	
Distance of tumor from anal verge, cm	5.5 (2.2)	6 (3.6)	0.8^e^
Tumor size, cm	4.1 (1.4)	3.7 (1.2)	0.332^c^
Surgical technique			0.375^b^
Laparoscopic surgery	24 (82.8%)	31(73.8%)	
Robotic surgery	5 (17.2%)	11 (26.2%)	
Pathological T stage			0.361^b^
T1	2 (6.9%)	8 (19%)	
T2	6 (20.7%)	7 (16.7%)	
T3	14 (48.3%)	14 (33.3%)	
T4	7 (24.1)	13 (31%)	
Pathological N stage			0.626^b^
N0	14 (48.3%)	25 (59.5%)	
N1	10 (34.5%)	12 (28.6%)	
N2	5 (17.2%)	5 (11.9%)	
Pathological M stage			—
M0	29 (100%)	42 (100%)	
Pathological TNM stage			0.317^b^
I	5 (17.2%)	14 (33.3%)	
II	10 (34.5%)	11 (26.2%)	
III	14 (48.3%)	17 (40.5%)	

a Values are shown as *n* (%) or mean (SD).

b Pearson’s χ2 test.

c Student’s *t*-test.

c Pearson’s χ2 test.

d Fisher’s exact test.

e Mann–Whitney *U* test.

ASA, American Society of Anesthesiologists; DSM, dumpling suture method; LAR-DLI, laparoscopic anterior rectal resection plus diverting loop ileostomy; TSM, traditional suture method.

### Intraoperative details and early postoperative outcomes of surgery

The enlarged initial incision length was 6.6 cm (1.0, SD) for the DSM group and 6.5 cm (1.0, SD) for the TSM group (*P*=0.447) (Table 2). Following suturing by two distinct methods, the final stoma size in the DSM and TSM group reduced to 2.9 cm (0.3, SD) and 3 cm (0.3, SD), respectively (*P*=0.152). No significant difference in incision dimensions was observed between the two groups before and after suturing. The operative time of stoma suture for DSM was not significant longer than that for the TSM method (10.9 min for DSM vs. 10.1 min for TSM, *P*=0.119). Additionally, the postoperative outcomes of time to first flatus, time to first defecation, time to first eat, postoperative intestinal obstruction, anastomotic leakage, renal insufficiency, and abdominal infection were similar between the groups (Table 2). However, the postoperative hospital stays in the DSM group was significantly shorter than that in the TSM group (8.4 days vs. 9.6 days, *P*=0.011).

**Table 2:** Intraoperative details and early postoperative outcomes of surgery^a^.

	Patients undergo LAR-DLI (*n*=71)		
Variable	DSM (*n*=29)^b^	TSM (*n*=42)^b^	*P*
Initial incision length, cm	6.6 (1.0)	6.5 (1.0)	0.447^c^
Length of final stoma, cm	2.9 (0.3)	3 (0.3)	0.152^c^
Operative time of stoma suture, min	10.9 (2.1)	10.1 (1.7)	0.119^d^
Time to first flatus, days	2.7 (0.8)	2.6 (1.1)	0.576^c^
Time to first defecation, days	3.1 (0.6)	3.2(1.2)	0.654^c^
Time to first eat, days	3.3 (1.5)	3.5 (1.3)	0.606^c^
Postoperative hospital stays, days	8.4 (1.5)	9.6 (2.1)	0.011^c^,^*^
Postoperative Intestinal obstruction			0.673^e^
Yes	2 (6.9%)	3 (7.1%)	
No	27 (93.1%)	39 (92.9%)	
Postoperative anastomotic leakage			0.64^e^
Yes	1 (3.4%)	3 (7.1%)	
No	28 (96.6%)	39 (92.9%)	
Postoperative renal insufficiency			0.784^e^
Yes	1 (3.4%)	2 (4.8%)	
No	28 (96.6%)	40 (95.2%)	
Postoperative abdominal infection			0.32^e^
Yes	2 (6.9%)	6 (14.3%)	
No	27 (93.1%)	36 (85.7%)	

*P*<0.05 was considered statistically significant.

a Early postoperative period refers to the period from the time of surgery to the time of discharge from hospital.

b Values are shown as *n* (%) or mean (SD).

c Student’s *t*-test.

d Mann–Whitney *U* test.

e Fisher’s exact test.

* *P*<0.05.

DSM, dumpling suture method; LAR-DLI, laparoscopic anterior rectal resection plus diverting loop ileostomy; TSM, traditional suture method.

### Stoma-related complications 1 month after surgery

Within 1 week following DSM surgery, the folded skin rapidly healed and flattened, forming a close attachment to the stoma, without skin elevation or fecal leakage. Stoma bag adhered well to the skin. One month after the surgery, the peristomal skin in the DSM group exhibited favorable healing, and no skin folds were observable (Fig. 3B).

One month postoperatively, the total stoma-related complication rate in the DSM group was significantly lower than that in TSM group (10.3 vs. 35.7%, *P*=0.016) (Table 3). Owing to peristomal incision infections, the predominant complications in the TSM group were skin-related complications, including skin erosion or ulcer (10/42, 23.8%), fecal dermatitis (9/42, 21.4%), and mucocutaneous detachment (6/42, 14.3%) (Fig. 2B, Table 3). In contrast, all skin-related complications in the DSM group were significantly lower than in the TSM group, with only 1 out of 29 patients experiencing fecal dermatitis (1/29, 3.4%). Furthermore, the DET scale serves as an effective tool specifically designed for the assessment of peristomal skin lesion conditions, the DSM group exhibited notably lower DET scores in comparison to the TSM group (*P*=0.001). There was no difference in other stoma complications including stomal hernia, prolapse, retraction, and stenosis between the two groups. Notably, the incidence of parastomal hernia was low in both groups (3.4 and 4.9%, respectively), due to the early suturing to narrow the incision of peritoneal layer prior to stoma suture.

**Table 3:** Stoma-related complications 1 month after surgery.

	Patients undergo LAR-DLI (*n*=71)		
Variable	DSM (*n*=29)^a^	TSM (*n*=42)^a^	*P*
Stoma-related complications (Total)	3 (10.3%)	15 (35.7%)	0.016^b^,^*^
Fecal dermatitis	1 (3.4%)	9 (21.4%)	0.032^b^,^*^
Skin erosion or ulcer	0 (0%)	10 (23.8%)	0.005^b^,^*^
Mucocutaneous detachment	0 (0%)	6 (14.3%)	0.033^b^,^*^
Stomal hernia	1 (3.4%)	2 (4.9%)	1.0^c^
Stomal prolapse	0 (0%)	0 (0%)	—
Stomal retraction	0 (0%)	1 (2.4%)	1.0^c^
Stomal stenosis	1 (3.4%)	0 (0%)	0.408^c^
DET score^d^			0.001^e^,^*^
None (0)	28 (96.6%)	27 (64.4%)	
Mild (1–5)	1 (3.4%)	7 (16.6%)	
Moderate (6–10)	0 (0%)	8 (19%)	
Severe (11–15)	0 (0%)	0 (0%)	
NRS pain score			0.048^e^,^*^
None (0)	20 (69%)	21 (50.1%)	
Mild (1–3)	9 (31%)	17 (40.4%)	
Moderate (4–7)	0 (0%)	4 (9.5%)	
Severe (8–10)	0 (0%)	0 (0%)	
Quality of life scale for patients with stoma (COH-QOL-OQ)^f^	141.4 (16.8)	165.9 (31.4)	0.001^e^,^*^
Stoma closure interval time, weeks	22.4 (9.5)	20.1 (7.7)	0.264^g^

*P*<0.05 was considered statistically significant.

a Values are shown as *n* (%) or mean (SD).

b Pearson’s χ2 test.

c Fisher’s exact test.

d DET score: The parastomal skin assessment scale, which were summed from three assessment values consisting of skin discoloration, erosion, and tissue overgrowth, and translated into an ordinal variable with four categories: None: DET=0; Mild: DET=1–5; Moderate=5–10; Severe=11–15.

e Mann–Whitney *U* test.

f Patients provided ratings based on the impact of the ostomy on physiological, lifestyle, and psychological dimensions. With a maximum score of 430, higher scores indicate a more pronounced detrimental effect of the ostomy on quality of life.

g Student’s *t*-test.

* *P*<0.05.

CHO-QOL-OQ, City of Hope Quality of Life-Ostomy Questionnaire; DET, discoloration, erosion, and tissue overgrowth assessment scale; DSM, dumpling suture method; LAR-DLI, laparoscopic anterior rectal resection plus diverting loop ileostomy; NRS, numerical rating scale; TSM, traditional suture method.

In addition, the pain scores and quality of life scores in the DSM group were both lower than those in the TSM group (*P*=0.048 and 0.001, respectively). The stoma closure interval time was 22.4 weeks in the DSM group and 20.1 weeks in the TSM group, which was not significantly different (*P*=0.264) (Table 3).

### Multivariate analysis for stoma-related complications of DLI

Multivariate regression analysis was conducted for factors associated with stoma-related complication. The following variables, which have the potential to influence stomal complications, were used to construct the model: age, sex, ASA class, BMI, combined diabetes, hypoalbuminemia, neoadjuvant therapy, initial incision length and surgical methods. DSM (OR: 0.131, 95% CI: 0.025–0.691, *P*=0.017) were found to be independent protective factors associated with stoma-related complications (Table 4). Combined diabetes was the only independent risk factor for the stoma-related complications (OR: 13.166, 95% CI: 1.52–114.047, *P*=0.019).

**Table 4:** Multivariate analysis for stoma-related complications of diverting loop ileostomy in laparoscopic anterior rectal resection with specimen extraction through stoma incision.

Variable	OR (95% CI)	*P*
Sex		
Male	0.637 (0.151–2.687)	0.54
Female	1 (Reference)	
Age	1.057 (0.982–1.138)	0.14
BMI	0.988 (0.774–1.261)	0.923
Combined diabetes		
Yes	13.166 (1.52–114.047)	0.019^*^
No	1 (Reference)	
ASA		
I	1 (Reference)	
II	3.115 (0.222–43.801)	0.399
III	0.390 (0.085–1.781)	0.224
Hypoalbuminemia		
Yes	1.214 (0.168–8.767)	0.847
No	1 (Reference)	
Neoadjuvant therapy		
Yes	1.826 (0.274–12.179)	0.534
No	1 (Reference)	
Initial incision length	1.52 (0.714–3.236)	0.277
Method of loop ileostomy		
DSM	0.131 (0.025–0.691)	0.017^*^
TSM	1 (Reference)	

*P*<0.05 was considered statistically significant.

* *P*<0.05.

ASA, American Society of Anesthesiologists; DSM, dumpling suture method; TSM: traditional suture method.

## Discussion

In LAR surgery, a DLI is usually performed in patients at high risk of anastomotic fistula (male, narrow pelvis, obesity, diabetes, anemia, severe underlying disease, poor bowel preparation, preoperative chemoradiotherapy, inflammatory bowel disease, poor anastomotic blood supply, high anastomotic tension, excessive number of cut closure strikes, etc.) to protect the anastomosis and reduce the subsequent complications^5^, which undergo closure surgery in 3–6 months.

Over the past decade, surgical techniques and instrumentation platforms have continued to improve and innovate, there has been a progressive reduction in the incision size for rectal surgery. The NOSES (Natural Orifice Specimen Extraction Surgery) procedure introduces a novel technique where the intestinal tract is exteriorized through body cavities, such as anus and vagina, and there is no surgical incision at all after the operation, which realizes minimally invasive to the greatest extent. In LAR-DLI surgery, many surgeons utilize an enlarged stoma incision for specimen extraction in an attempt to avoid an auxiliary abdominal incision^6^. Strictly speaking, specimen extraction through stoma incision (EXSI) surgery does not belong to NOSES, yet postoperatively, it shares the characteristic of no additional auxiliary incisions in the abdominal wall. With the advantages of reduced trauma, accelerated recovery and enhanced postoperative cosmetics, the EXSI surgery has gained widespread and significant traction in rectal surgery.

However, this surgical approach is associated with a higher risk of stoma-related complications, with a reported incidence of 13–33%, significantly higher than that of traditional ostomy, thereby being doubted and limited in widespread use^6–9^. In this study, the incidence of stomal complication in the TSM group was 35.7% (Table 3). These complications were predominantly skin-related, including skin erosion or ulcer (10/42, 23.8%), fecal dermatitis (9/42, 21.4%) and mucocutaneous detachment (6/42, 14.3%). The principal factor contributing to stomal complications was infection of peristomal incision, subsequently leading to fecal dermatitis and mucocutaneous detachment. Extraction of specimens through the incision of ileostomy leads to the generation of relatively extensive incisions (5–10 cm). To reduce the incision size and match it to the stoma size, TSM uses interrupted suture at both ends of the incision to reduce the size to 3 cm, followed by fixing the stoma to the skin (Fig. 3C). The TSM; however, results in the formation of incisions adjacent to the stoma, whereby the highly corrosive intestinal fluids can readily cause infection upon direct contact with the peristomal incision. Consequently, it increases the risk of peristomal skin infection, subsequently leading to the development of fecal dermatitis, skin erosion, or ulcer and mucocutaneous detachment.

With the aim of reducing stoma-related complications and improving the EXSI procedure, we propose the DSM (Fig. 3C). As shown in Table 3, the stomal complication rate in DSM group was 10.3% (3/29) and lower than 35.7% (15/42) in TSM group (*P*=0.016), and the DSM was an independent protective factor for stoma-related complications (OR: 0.131, 95% CI: 0.025–0.691, *P*=0.017). In addition, DSM also significantly reduced DET scores (*P*=0.001), suggesting that DSM can effectively protect the peristomal skin and reduce skin-related complications, including skin erosion or ulcer (0 vs. 23.8%, *P*=0.005), fecal dermatitis (3.4 vs. 21.4%, *P*=0.016) and mucocutaneous detachment (0 vs. 14.3%, *P*=0.033). However, there were no differences in nonskin-related stoma complications between the two groups, including stomal hernia (3.4 vs. 4.9%), stomal prolapse (0 vs. 0%), stomal retraction (0 vs. 2.4%), and stomal stenosis (3.4 vs. 0%), indicating that the primary advantage of DSM lies in peristomal skin protection. This phenomenon might be attributed to progressive reduction of the incision using the folded skin suture in DSM. Postoperatively, there are no peristomal incisions, thereby effectively preventing skin infections. Within 1 week following the surgery, the folded skin rapidly healed and flattened, forming a close attachment to the stoma without interfering with the adherence of the stoma bag.

Moreover, we evaluated the safety of DSM. As shown in Table 2, there were no differences in operative time, time to first flatus, time to first defecation, time to first eat, postoperative complications (intestinal obstruction, anastomotic leakage, renal insufficiency, and abdominal infection) and stoma closure interval time between the two groups, indicating that DSM does not increase the risk of surgery and is safe and feasible. The length of postoperative hospital stays for patients undergoing DSM was shorter than that for TSM (8.4 days vs. 9.6 days, *P*=0.011), suggesting that DSM may expedite postoperative recovery. This phenomenon could potentially be attributed to stomal infections in the TSM group, which delayed patient discharge.

There are some tips for the DSM technique. First, the rubber tube is not necessary in either TSM or DSM procedures; it only serves to support the stoma and prevent stoma retraction. In cases of well-fitted and well-anchored stoma, the absence of rubber tube does not increase the risk of stomal complications. Second, since the incision of the peritoneal layer is also relatively large, in order to prevent parastomal hernia, both DSM or TSM should suture the ends of the peritoneal layer to reduce its size before suturing the stoma. Third, the number of skin-folded sutures should be determined based on the size of the original incision, typically ranging from 8 to 12 sutures. If the original incision is larger, the number of sutures should correspondingly increase, and the final length of the incision should be reduced to about 3 cm.

It is worth noting that, in TSM procedure, there are different ways to open the stoma in the final approach. Following the opening of the stoma wall, many surgeons proceed to circumferentially fix both the proximal and distal stoma walls to skin incision with external flap suture. Regardless of the different surgical approach employed in TSM, the presence of peristomal incisions is unavoidable, leading to higher risk of peristomal skin infections and stomal complications. When employing DSM procedure, we do not recommend suture the cutting edge of opened ileum to the skin, as it increases surgical procedures and raises the risk of additional stomal complications. A 1–2 cm incision in the intestinal wall to simply open the stoma is sufficient. Following surgery, intestinal fluid drained from the proximal opening of the ostomy, without refluxing into the distal opening.

Our study has some limitations. First, it is a retrospective analysis with limited cases; therefore, it has some inherent biases. Although no significant differences were detected at baseline demographics among the two groups, it was still impossible to control for differences in patient selection. In order to further confirm the results, we are currently conducting a prospective randomized controlled trial of these two methods of DLI, and have registered for clinical trial. Second, we evaluated the stoma complications only at 1 month postoperatively, and did not evaluate the outcomes within 7 days postoperatively as well as in longer term. This evaluation was conducted because the differences of stomal complications between the two groups are most evident in the first month postoperatively. Third, the DSM procedure is applicable in cases where the stoma incision is large and needs to be reduced, especially for LAR-DLI with specimen extraction through stoma incision. Moreover, in loop transverse colostomy, DSM can also be utilized to reduce the incision size when the stoma incision is relatively large. In the case of standard DLI with specimen extraction through auxiliary skin incision, there is no need to employ the DSM or TSM to reduce the stoma incision size, as it is not enlarged. The stomal complication rate of traditional DLI reported in the literature vary widely, ranging from 10 to 80%^11–16^. The stomal complication rate of DSM reported in our study was 10.3%. In the case of specimen extraction through auxiliary skin incision (not stoma incision), dumpling suture does not increase incidence of stomal complications; however, its advantages may also not be fully realized.

## Conclusions

Specimen extraction through stoma incision increased the risk of peristomal skin infections and stomal complications with enlarged stomal incision. DSM utilizes a method of skin folding suture to reduce the enlarged incision size, without creation of peristomal incisions, which is safe and effective in preventing peristomal skin infections and reducing the incidence of stomal complications. This procedure offers a novel suturing approach for loop ileostomy with enlarged incision, effectively reducing the postoperative trauma and incidence of stomal complications.

## Ethical approval

The study was approved by Ethics Committee of Xinhua Hospital affiliated to Shanghai Jiao Tong University School of Medicine (XHEC-2023-D-146) and was carried out in accordance with the 1964 Helsinki Declaration.

## Consent

Written informed consent was obtained from the patient for publication and any accompanying images. A copy of the written consent is available for review by the Editor-in-Chief of this journal on request.

## Sources of funding

This work was supported by the National Natural Science Foundation of China (82273369), and Natural Science Foundation of Shanghai (21ZR1441400).

## Author contribution

J.G.: data curation, investigation, methodology, formal analysis, writing – original draft; J.W.: data curation, investigation, methodology, software, visualization; X.H., and W.D.: data curation and investigation; L.C. and P.D.: conceptualization, writing – review and editing; Z.L.: conceptualization, methodology, and visualization; T.W.: conceptualization, investigation, methodology, resources, project administration, writing – review and editing.

## Conflicts of interest disclosure

The authors declare that they have no known competing interests or personal relationships that could have appeared to influence the work reported in this paper.

## Research registration unique identifying number (UIN)

The trial was registered by the Clinical Trial of U.S. National Library of Medicine with a unique identification number (NCT06010043).

## Guarantor

Tingyu Wu.

## Data availability statement

Data used for this research are available from corresponding author upon request. All authors confirm its originality and bear responsibility of postproduction queries.

## Provenance and peer review

Not commissioned, externally peer-reviewed.

## Supplementary Material

SUPPLEMENTARY MATERIAL
